# A transcriptional network of cell cycle dysregulation in noninvasive papillary urothelial carcinoma

**DOI:** 10.1038/s41598-022-20927-9

**Published:** 2022-10-03

**Authors:** Joshua I. Warrick, Margaret A. Knowles, Carolyn D. Hurst, Lauren Shuman, Jay D. Raman, Vonn Walter, Jeffrey Putt, Lars Dyrskjøt, Clarice Groeneveld, Mauro A. A. Castro, A. Gordon Robertson, David J. DeGraff

**Affiliations:** 1 Department of Pathology and Laboratory Medicine, Pennsylvania State University College of Medicine, 500 University Drive, Hershey, PA 17033 USA; 2 Department of Urology, Pennsylvania State University College of Medicine, Hershey, PA 17033 USA; 3 Divison of Molecular Medicine, Leeds Institute of Molecular Research at St James’s, St James’s University Hospital, Beckett Street, Leeds, LS9 7TF UK; 4 Department of Public Health Sciences, Pennsylvania State University College of Medicine, Hershey, PA 17033 USA; 5 Department of Biochemistry and Molecular Biology, Pennsylvania State University College of Medicine, Hershey, PA 17033 USA; 6 Department of Molecular Medicine, Aarhus University Hospital, Aarhus, Denmark; 7 Cartes d’Identité des Tumeurs (CIT) Program, Ligue Nationale Contre le Cancer, Équipe Oncologie Moleculaire, Institut Curie, Paris, France; 8 Bioinformatics and Systems Biology Laboratory, Federal University of Paraná, Curitiba, PR 81520-260 Brazil; 9 Bioinformatics, BC Cancer Agency, Vancouver, BC Canada

**Keywords:** Urological cancer, Bladder cancer

## Abstract

Human cancers display a restricted set of expression profiles, despite diverse mutational drivers. This has led to the hypothesis that select sets of transcription factors act on similar target genes as an integrated network, buffering a tumor’s transcriptional state. Noninvasive papillary urothelial carcinoma (NIPUC) with higher cell cycle activity has higher risk of recurrence and progression. In this paper, we describe a transcriptional network of cell cycle dysregulation in NIPUC, which was delineated using the ARACNe algorithm applied to expression data from a new cohort (n = 81, RNA sequencing), and two previously published cohorts. The transcriptional network comprised 121 transcription factors, including the pluripotency factors SOX2 and SALL4, the sex hormone binding receptors ESR1 and PGR, and multiple homeobox factors. Of these 121 transcription factors, 65 and 56 were more active in tumors with greater and less cell cycle activity, respectively. When clustered by activity of these transcription factors, tumors divided into High Cell Cycle versus Low Cell Cycle groups. Tumors in the High Cell Cycle group demonstrated greater mutational burden and copy number instability. A putative mutational driver of cell cycle dysregulation, such as homozygous loss of *CDKN2A*, was found in only 50% of High Cell Cycle NIPUC, suggesting a prominent role of transcription factor activity in driving cell cycle dysregulation. Activity of the 121 transcription factors strongly associated with expression of EZH2 and other members of the PRC2 complex, suggesting regulation by this complex influences expression of the transcription factors in this network. Activity of transcription factors in this network also associated with signatures of pluripotency and epithelial-to-mesenchymal transition (EMT), suggesting they play a role in driving evolution to invasive carcinoma. Consistent with this, these transcription factors differed in activity between NIPUC and invasive urothelial carcinoma.

## Introduction

There are over 500,000 new cases of bladder cancer diagnosed annually worldwide^1^. Seventy percent are diagnosed as noninvasive papillary urothelial carcinoma (NIPUC)^2^, an early state in tumor evolution referred to clinically as *stage Ta*. These are challenging to manage. Patients may be treated conservatively, with transurethral resection of the tumor, often followed by intravesical instillation of Bacillus Calmette–Guérin (BCG)^3^. However, the majority recur^4^, and a subset progresses to advanced-stage disease^2^, which is often fatal. BCG also confers considerable morbidity, including bladder pain, voiding dysfunction, and occasional sepsis^5^. Patients may alternatively be managed definitively with radical cystectomy, which reduces risk of death from disease compared with conservative management^6^. However, this treatment profoundly impacts urinary function. Management of NIPUC is thus a difficult balance between preventing mortality and preserving quality of life. Better treatments and prognostic tests are needed.

Human cancers tend to take on a relatively small set of expression profiles, despite having diverse mutational drivers^7,8^. This has led to the hypothesis that there exists a universal gene-regulatory architecture, in which a small number of master regulator proteins, such as transcription factors (TFs), initiate and maintain these recurrent transcriptional states, and thereby govern tumor behavior^9^. These states are diverse, including signatures of drug sensitivity and risk of cancer progression, among others^9^.

NIPUC is diverse in gene expression, particularly cell cycle activity, and many studies have shown worse clinical outcomes in early-stage bladder cancers with greater cell cycle dysregulation (reviewed in^10^). In the largest genomic study to date, tumors expressed either genes involved in the late cell cycle, such as Forkhead Box M1 (*FOXM1)*, or genes involved in the early cell cycle, such as Cyclin D1 (*CCND1*)^11,12^. These transcriptional states predicted outcome, with those in the late cell cycle group having higher risk of stage progression. Strikingly, tumors with activation of the late cell cycle consistently expressed other signatures, distinct from the cell cycle, such as signatures of cancer stem cells and epithelial-to-mesenchymal transition (EMT)^11^. This finding suggests that a transcriptional network operates in these late cell cycle tumors, in which TFs induce biological attributes that drive aggressive tumor behavior. If such a transcriptional network were delineated, it could be used to identify druggable targets in early-stage bladder cancer, and develop tests that predict outcomes, improving treatment options in this challenging disease.

In this study, we identified a network of transcriptional factors whose activity strongly associates with cell cycle activity in NIPUC. TFs in this network were diverse, including pluripotency and homeobox factors. Activity of these TFs associated with signatures of pluripotency and EMT, as well as expression of members of the Polycomb Repressor Complex 2 (PRC2). The findings suggest TFs in this network may drive aggressive behavior in NIPUC through activation of pluripotency pathways.

## Results

### Cohorts

Our study was retrospective and utilized three cohorts of NIPUC. These were used to develop the transcriptional network and test its relationship with biological features, such as pathway activity. Cohort 1 consisted of NIPUC on which whole transcriptome RNA sequencing and whole exome DNA sequencing were newly performed, specifically for this study (n = 81, RNA sequencing; DNA sequencing performed on 36 of these). Cohort 2 consisted of cases of NIPUC from the UROMOL study, for which RNA sequencing-based expression data are publicly available (n = 397)^12^. Cohort 3 consisted of cases of NIPUC from Leeds University, for which expression data by Affymetrix microarray are publicly available (n = 107, see “Materials and methods”).^13^

We also compared activity of the transcriptional network between NIPUC and invasive urothelial carcinoma. This included both invasive cancers limited to the lamina propria and those invading the muscularis propria. This was performed using lamina propria-invasive cancers from the UROMOL (n = 135)^12^ and Leeds (n = 102) Cohorts, and MIBC from The Cancer Genome Atlas (TCGA) cohort^14^.

For clarity, we use the terms “Cohort 1,” “Cohort 2,” and “Cohort 3” to describe groups that consist entirely of NIPUC. We use the terms “UROMOL Cohort” and “Leeds Cohort” to describe groups of cases with both NIPUC and lamina propria-invasive cancer. Clinical details of the three cohorts are presented in Table S1.

### A transcriptional network of cell cycle dysregulation in noninvasive papillary urothelial carcinoma

Many studies have shown that NIPUC with greater cell cycle dysregulation has higher risk of recurrence and progression to advanced-stage disease (reviewed in^10^). We thus sought to identify a network of TFs active in cell cycle dysregulation of NIPUC, to better understand the transcriptional changes that promote recurrence and prime tumors to invade.

We first generated a transcriptional regulatory network from NIPUC expression data using the ARACNe algorithm, a method that takes a list of curated TFs and identifies target genes likely regulated by each of them, using mutual information between expression of a given TF and potential target genes^14–17^. The targets of each TF are termed its “TF regulon.” We used a list of 1639 curated TFs^18^. We then identified TF regulons whose activity associated with activity of the cell cycle. This involved a step-wise approach applied to Cohorts 1 and 2, with orthogonal measures of cell cycle activity, and two layers of statistical tests applied to each cohort (details of the entire approach are shown Fig. S1 and “Materials and methods”). Measures of cell cycle activity were mitotic index^4^, defined as the number of mitotic figures seen histologically in 10 high-powered microscopic fields^4^, and expression of late versus early cell cycle genes, with expression of the former considered to indicate greater cell cycle activity^11^. Activity of each TF regulon was quantified in each tumor as “enrichment score”. This is a continuous variable reflecting expression of targets within each TF regulon, determined using single sample gene set enrichment analysis (ssGSEA), with enrichment scores ranging from − 2 to 2 (positive scores indicate high enrichment, and negative scores indicate low enrichment)^15^.

**Figure 1:**
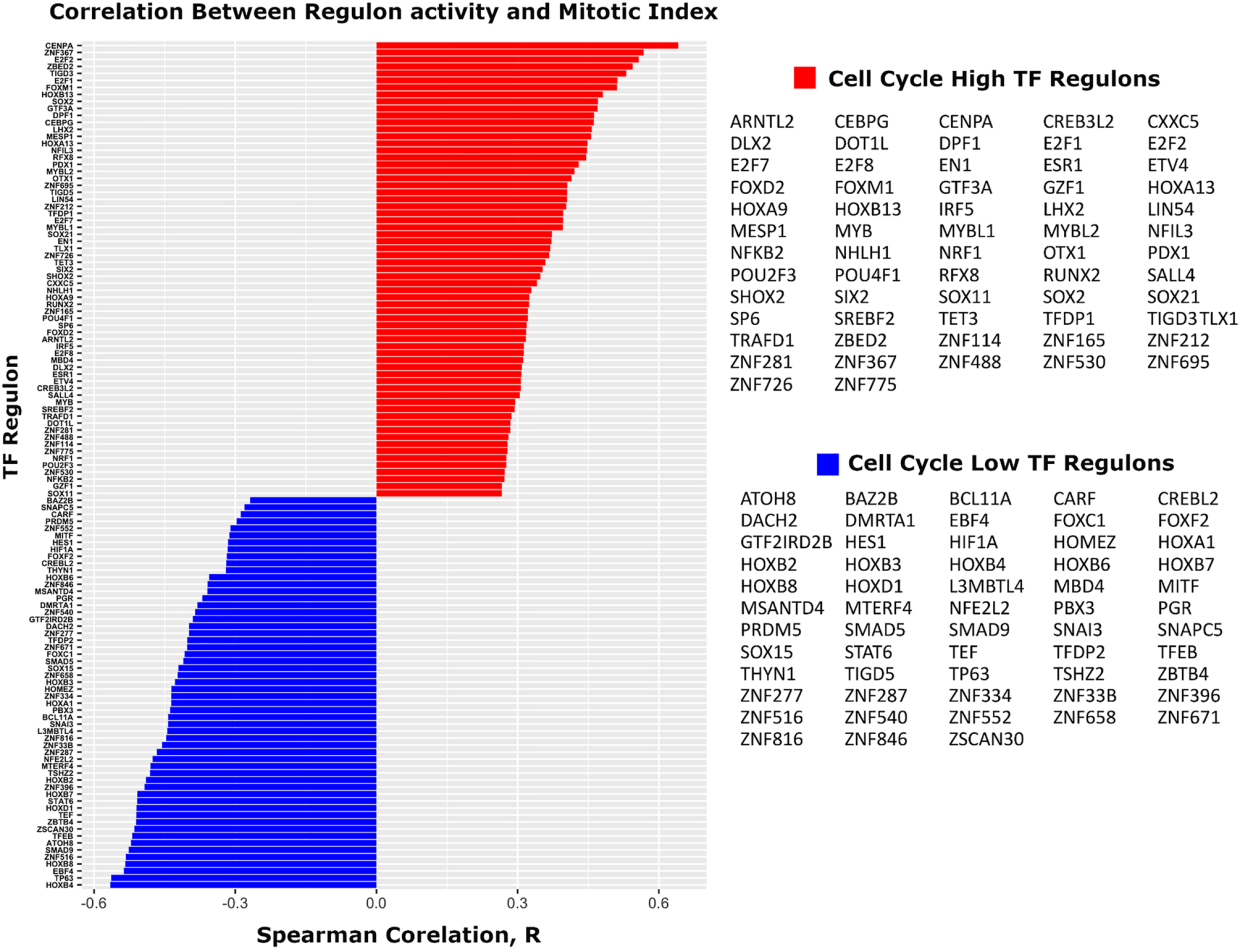
Transcription factor (TF) regulons associated with cell cycle activity. Activity of 121 TF regulons significantly associated with cell cycle activity. Of these, 65 TF regulons associated with high cell cycle and 56 associated with low cell cycle activity. In addition to TFs with a known role in the cell cycle, significant TF regulons include the pluripotency factors SALL4 and SOX2, multiple homeobox factors, and the sex hormone binding receptors ESR1 and PGR. The bar plots demonstrate the correlation between mitotic index and activity of each significant TF regulon, determined using Cohort 1.

This approach showed 121 TF regulons differed in activity between early and late cell cycle groups and associated with mitotic index (Wilcoxon rank sum test and Spearman correlation, respectively, q < 0.05 considered significant). Of these, 65 associated with high cell cycle activity, and 56 associated with low cell cycle activity. Significant TF regulons are shown in Fig. 1. All TF regulons whose activity positively correlated with mitotic index were more active in late cell cycle group, and vice versa. Notably absent from these selected regulons were many TFs known to urothelial differentiation, such as FOXA1, GATA3, and PPARG^19^. TF regulons, including TFs and all targets, are shown in Table S2.

As anticipated, tumors with greater cell cycle activity demonstrated greater activity of TF regulons with a known role in regulating the cell cycle, including FOXM1, E2F Transcription factors 1 and 2 (E2F1, E2F2), and M Proto-oncogene (MYB). Tumors with greater cell cycle activity also showed greater activity of TF regulons not classically associated with the cell cycle. These included TFs of pluripotency and embryonic development, such as Spalt Like Transcription Factor 4 (SALL4) and SRY-BOX Transcription Factor 2 (SOX2), as well as three members of the Homeobox (HOX) gene family, a group of TFs with a central role in embryonic development. Other notable factors associated with high cell cycle activity included the POU class homeobox factors POU2F3 and POU4F1, and the homeobox factor SHOX2. Tumors with lesser cell cycle activity showed greater activity of eight HOX factors, a striking finding considering there are only 39 HOX genes in humans, indicating disproportionate activity of this family in NIPUC. Lesser cell cycle tumors also showed greater activity of the melanocyte factor MITF, the immune factor STAT6, and the hypoxia factor HIF1A.

Two sex hormone binding factors were also identified: ESR1, whose activity associated with high cell cycle activity, and PGR, whose activity associated with low cell cycle activity. Activity of these factors did not differ between male and female patients (p = 0.1 and p = 0.2, respectively; Wilcoxon rank sum test, Cohort 1). Several members of the FOX, SOX, and ZNF family of transcription factors were also significant, in both the low and high cell cycle groups.

To validate these findings, we next quantified activity of the 121 TF regulons in NIPUC from Cohort 3. Of these, there was sufficient expression data to quantify activity of 112 TF regulons; 88% of these differed in activity between early and late cell cycle groups, all in the expected direction, corroborating the above findings (q < 0.05, Wilcoxon rank sum test, Table S3).

Late cell cycle tumors were enriched in high-grade tumors in Cohort 1 (p < 0.001, Fisher’s exact test), but not in Cohort 2 (p = 0.5, Fisher’s exact test). Late cell cycle tumors had greater mitotic index than early cell cycle tumors in Cohort 1 (p < 0.001, Wilcoxon rank sum test). The late versus early cell cycle tumors did not differ in risk of recurrence or progression in any cohort (all p > 0.1, logrank test, all three cohorts; progression not tested in Cohort 3 because only one patient experienced progression).

### TF regulon activity and signatures of cellular signaling, pluripotency, and epithelial-mesenchymal transition

**Figure 2:**
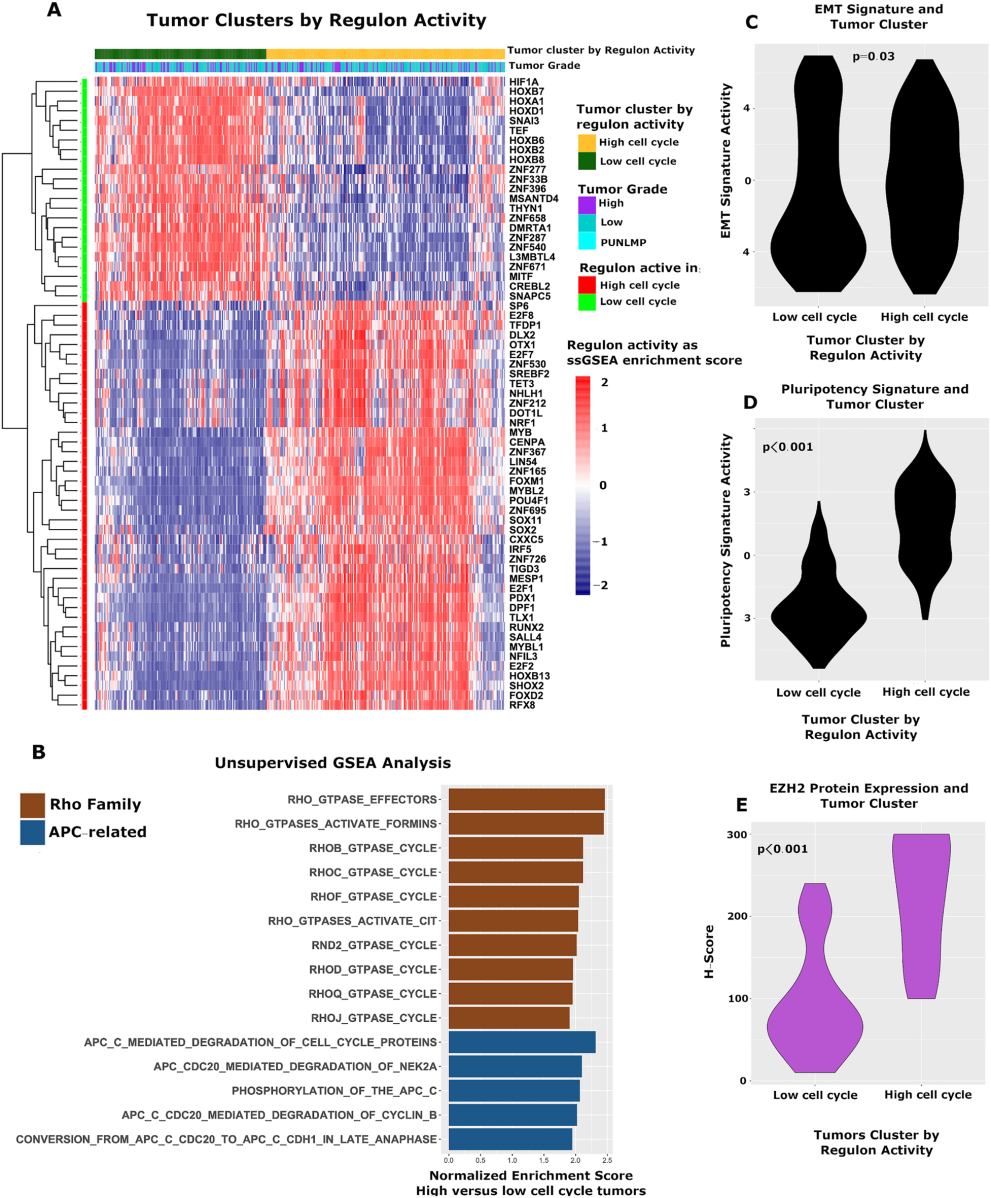
Activity of TF regulons and activity of cellular pathways, EMT, and pluripotency signatures. (**A**) Consensus clustering divided tumors into High Cell Cycle and Low Cell Cycle groups, which differed in regulon activity. (**B**) GSEA demonstrated tumors in the High Cell Cycle group had greater activity of multiple signatures, most notably signatures in the Rho family and APC pathway. Tumors in the High Cell Cycle group demonstrated greater activity of established EMT (**C**) and Pluripotency (**D**) signatures^20,21^, and expressed higher levels of the PRC2 member EZH2 at the protein level by immunohistochemistry (**E**). (**A**–**D** from Cohort 2, **E** from Cohort 1).

We next sought to identify the relationships between activity of the cell cycle and activity of cellular pathways. Given the focus of the study is TF regulon activity, we defined cell cycle activity based on regulon activity for this and remaining portions of the analysis. Specifically, we clustered tumors by TF regulon activity, which divided them into two large groups, one with greater activity of high cell cycle regulons, the other with greater activity of low cell cycle regulons. We termed these the “High Cell Cycle” and “Low Cell Cycle” clusters, respectively (Fig. 2A). Unsupervised pathway analysis using GSEA and Reactome signatures showed 14% of evaluated pathways significantly differed in activity between the High Cell Cycle and Low Cell Cycle clusters (116 of 807 signatures; q < 0.05; Table S4). As expected, signatures involved in the cell cycle/DNA repair dominated, accounting for 56% of all signatures. Also notable were signatures of Rho family and Adenomatous polyposis coli (APC) signaling, all of which were more active in the High Cell Cycle cluster (Fig. 2B).

Because many of the significant TFs have known roles in cellular differentiation and pluripotency, we next investigated the association between activity of these TF regulons and signatures of EMT and pluripotency, using ssGSEA and established genes lists^20,21^. This showed the High Cell Cycle cluster had greater activity of both the EMT signature (p = 0.03, Wilcoxon rank sum test; Fig. 2C) and the pluripotency signature (p < 0.001, Wilcoxon rank sum test; Fig. 2D). The High Cell Cycle cluster likewise demonstrated greater expression of three of the five core EMT transcription factors^22^ namely *SNAI1* (p < 0.001, Wilcoxon rank sum test), *ZEB1* (p = 0.003, Wilcoxon rank sum test), and *ZEB2* (p < 0.001, Wilcoxon rank sum test), as shown Fig. S2. *TWIST1* and *SNAI2* were non-significant (p = 0.06 and 0.1, respectively, Wilcoxon rank sum test).

Prior studies have shown a high fraction of bladder cancers express the methyltransferase EZH2, a key component of the PRC2 complex, a protein complex with a central role in pluripotency and embryogenesis^23^. Consistent with this, tumors in the High Cell Cycle cluster demonstrated greater expression of EZH2 at the protein (p < 0.0001, Wilcoxon rank sum test; Fig. 2E, Fig. S3; quantified by immunohistochemistry; Cohort 1) and mRNA levels (p < 0.001, Wilcoxon rank sum test; Cohort 2). Tumors in the High Cell Cycle cluster also expressed higher levels of the other members of the PRC2 complex, specifically *SUZ12* (p < 0.001, Wilcoxon rank sum test; Cohort 2), *EZH1* (p < 0.001, Wilcoxon rank sum test; Cohort 2), and *EED* (p = 0.03, Wilcoxon rank sum test; Cohort 2), at the mRNA level (Fig. S4).

We next compared members of these genes lists to those in the early versus late cell cycle gene lists used to define identify TF regulons associated with cell cycle activity, and found that only 5% of genes in the pluripotency signature were in the late cell cycle gene list (*CDC25A* and *MYBL2*) and none of the genes in the EMT or PRC2 genes lists were in the early or late cell cycle gene lists.

Lindskrog et al. recently reported an expression-based molecular classifier of early-stage bladder cancer, which assigned Classes 1, 2a, 2b, or 3, with those in the Class 2 groups having the highest risk of stage progression^12^. Consistent with this, tumors in the High Cell Cycle cluster tended to be Class 2a (p < 0.0001, Fisher’s exact test, Cohort 2, Fig. S5).

### Dual TF regulon analysis identifies two groups of TFs that oppose one another

**Figure 3:**
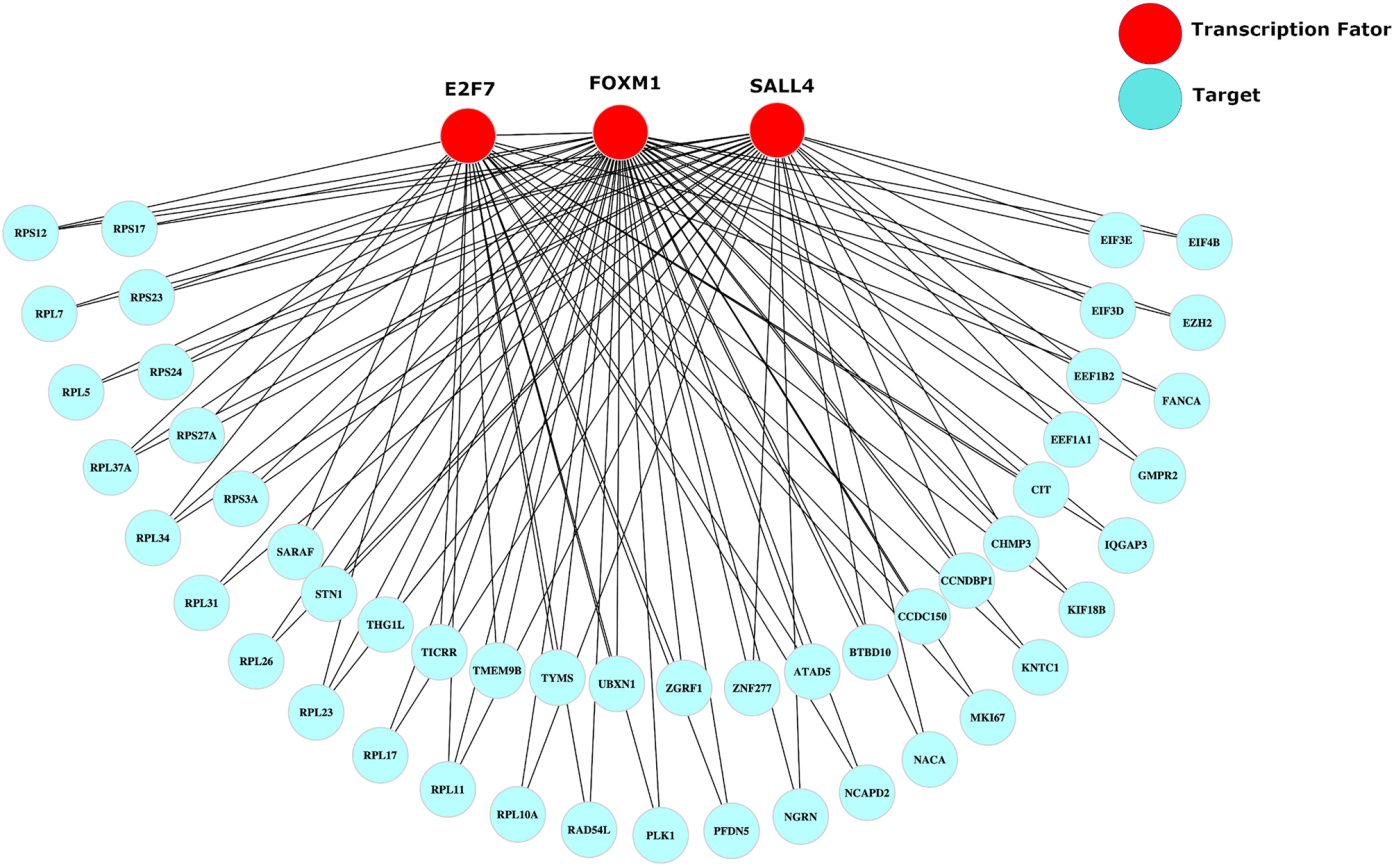
Example portion of the transcriptional network. A small portion of the transcriptional network is shown, including targets of E2F1, FOXM1, and SALL4 shared by at least two of these transcription factors. There was great overlap in targets, including genes involved in the cell cycle, such as *MKI67* and *CCNDBP1*.

**Figure 4:**
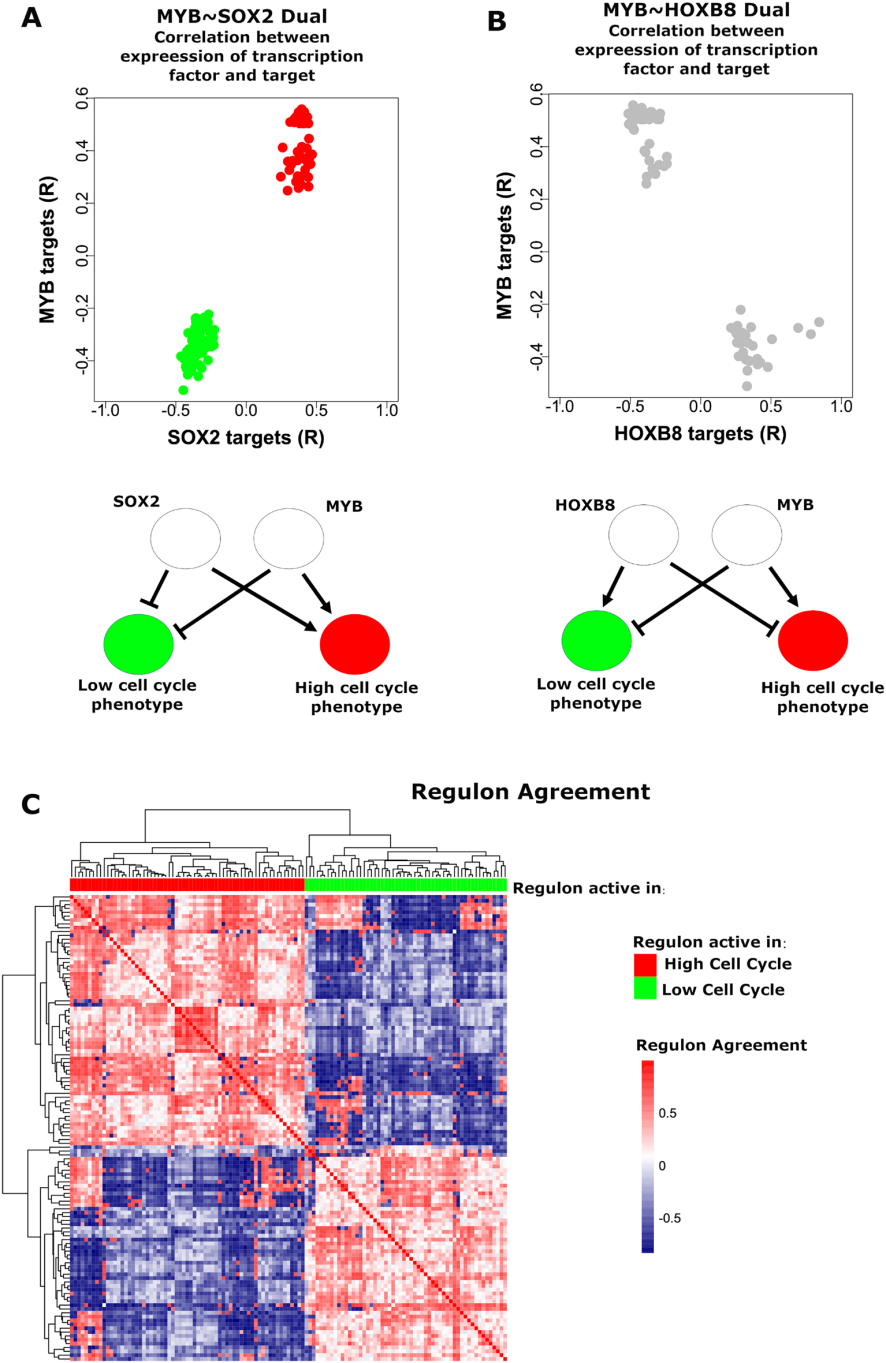
Dual regulons analysis. Two TF regulon duals are selected for illustration, (**A**) MYB ~ SOX2 and (**B**) MYB ~ HOXB8. For each, a dot plot demonstrates the correlation (R) between expression of each TF and each of its targets, for all targets shared by the TF pair (i.e. each dot corresponds to a target shared by the two TFs, and the value is the correlation between expression of the target and the given TF). Red dots indicate positive correlation, blue dots indicate negative correlation, and grey dots indicate opposite correlation. A schematic is also shown for each, demonstrating the effect each TF putatively has on the phenotype, arrows indicating stimulation and flat bars indicating inhibition. MYB and SOX2 both appear to positively regulate the High Cell Cycle phenotype and negatively regulate the Low Cell Cycle phenotype, while MYB and HOXB8 appear to have opposite effects. (**C**) TF regulons were clustered by Regulon agreement, the Spearman correlation between TF pairs and their relationship with target genes (i.e. correlation based on data points in **A** and **B**), which divided TF regulons into two clusters, one enriched in regulons of high cell cycle activity, the other enriched in regulons of low cell cycle activity. Figures generated using expression data from Cohort 2.

Transcriptional network analysis is valuable in part because it identifies TFs that act on similar targets, providing insight into redundancy of their activity, a feature relevant to development of drugs and diagnostic tests. For example, in our analysis there was overlap in the targets of FOXM1, SALL4, and E2F7, including targets with a known role in the cell cycle, such as *MKI67* and *CCNDBP1* (Fig. 3). To further investigate similarity in targets genes among TF regulons, we utilized the RTNduals extension of the RTN package^15,16,24^ to perform dual regulon analysis. This procedure identifies pairs of TF regulons that potentially co-activate or co-repress shared target genes, as well as pairs of TFs that potentially compete, influencing targets in opposite directions^24^. Using NIPUC from Cohort 2, and isolating analysis to the 121 TF regulons identified above, we found 84 TF regulons were involved in at least one dual regulon, and found 243 significant dual regulon associations in total (Table S5). For example, MYB and SOX2 had similar targets and the same effect on them, suggesting both promote the High Cell Cycle phenotype (Fig. 4A). In contrast, MYB and HOXB8 had similar targets but the opposite effect on them, suggesting these promote the High versus Low Cell Cycle phenotypes, respectively, by acting on the same set of genes in an antagonistic manner (Fig. 4B). We then generated a correlation matrix, based on “Regulon agreement,” defined as the correlation between TF pairs and their relationship with target genes (i.e. correlation based on data points such as those shown in Fig. 4A and B with MYB and SOX2 positively correlated, and MYB and HOXB8 negatively correlated). This largely divided TFs into two groups, which mirrored activity of their regulons in the High versus Low Cell Cycle clusters (Fig. 4C). These two groups of TFs thus appear to act on similar sets of target genes but have opposite effects on them, suggesting these groups oppose one another.

### Mutational changes and TF regulon activity

**Figure 5:**
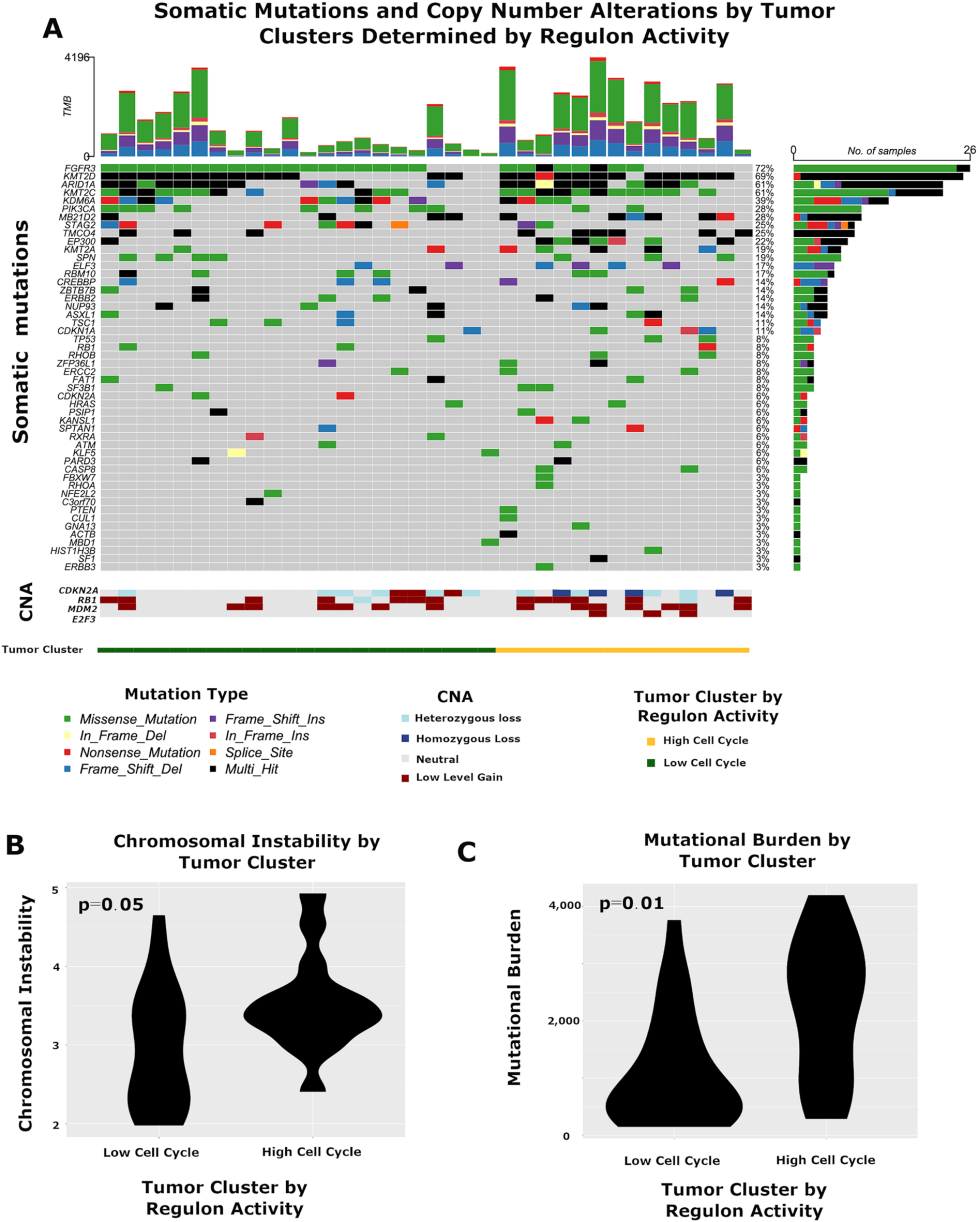
Somatic mutations and copy number alterations on non-invasive papillary urothelial carcinoma. (**A**) Tumors demonstrated somatic alterations common in noninvasive papillary urothelial carcinoma, including somatic mutations in *FGFR3*, *STAG2*, and multiple chromatin remodeling genes, such as *ARID1A* and *KMT2D*, as well as homozygous and heterozygous copy number losses of *CDKN2A* (CNA = copy number alterations). Tumors in the High Cell Cycle group demonstrated (**B**) greater chromosomal instability and (**C**) higher tumoral mutational burden. Chromosomal instability was defined as the mean of the chromosome-level segmentation means in each tumor. Tumor mutation burden was defined as the total number of somatic mutations per tumor after filtering. All data derived from Cohort 1.

To investigate the relationship between regulon activity and somatic mutations in NIPUC, we performed whole exome sequencing on 36 cases from Cohort 1 (Fig. 5, Table S6). This identified high rates of mutation in *FGFR3*, *STAG3*, *PIK3CA*, and multiple chromatin remodeling genes, including *EP300*, KDM6A, KMT2A, KMT2C, KMT2D, and ARID1A, consistent with prior studies of NIPUC^25,26^. Mutations were also identified in genes active in cell cycle control, such *CDKN1A* and *RB1*. Copy number alterations were also common. For example, copy number loss of the tumor suppressor gene *CDKN2A* was frequent, with homozygous loss in 11% (4 cases) and heterozygous loss in 25% (9 cases), consistent with prior studies of NIPUC^27^. Cases with homozygous loss expressed lower levels of *CDKN2A* compared with cases lacking copy number loss (p = 0.01, Wilcoxon rank sum test; Fig. S6), while no difference in *CDKN2A* expression was seen with heterozygous loss (p = 0.5, Wilcoxon rank sum test). No case demonstrated homozygous loss of *RB1*, or high-level amplification of *MDM2* or *E2F3*, findings known to drive cell cycle dysregulation in bladder cancer more broadly^14^.

NIPUC in the High Cell Cycle cluster had higher tumor mutational burden (p = 0.01, Wilcoxon rank sum test) and greater chromosomal instability (p = 0.05, Wilcoxon rank sum test) compared with those in the Low Cell Cycle cluster (Figs. 5B,C), the latter finding consistent with findings by Hurst et al.^26^. (Chromosomal instability was defined as the mean of the chromosome-level segmentation means for each tumor, similar to prior studies^28^). All cases with homozygous loss of *CDKN2A* were in the High Cell Cycle group, accounting for 29% of High Cell Cycle tumors. Mutation in *EP300* was more common in High Cell Cycle tumors (q = 0.05, Fisher’s exact test), but the Regulon clusters did not differ statistically in mutational rate of any other gene. Mutations in genes known to regulate the cell cycle (e.g. *CDKN2A, RB1, CDKN1A*) were identified in both the High Cell Cycle and Low Cell Cycle groups. Of the High Cell Cycle cases, only 50% harbored homozygous loss of *CDKN2A* or any mutation in a gene that directly regulates the cell cycle.

### Activity of TF regulons in NIPUC versus invasive bladder cancer

To elucidate the role the 121 TF regulons play in progression to invasive bladder cancer, we next compared activity of these regulons in NIPUC to both lamina propria-invasive and muscle-invasive bladder cancer (MIBC). We noted the associations between activity of these TF regulons and signatures of EMT and pluripotency, as shown in Fig. 2. We also noted EMT and pluripotency likely play a role in tumor evolution from a noninvasive to an invasive phenotype^20,22^. We thus suspected a subset of the TF regulons drives evolution to the EMT and pluripotency phenotype in NIPUC. We likewise hypothesized activation of these biological features primes these noninvasive tumors to invade, and remains active in the resulting invasive carcinomas. We therefore hypothesized that activity of the high cell cycle TF regulons would be greater in invasive urothelial carcinoma compared with NIPUC.

**Figure 6:**
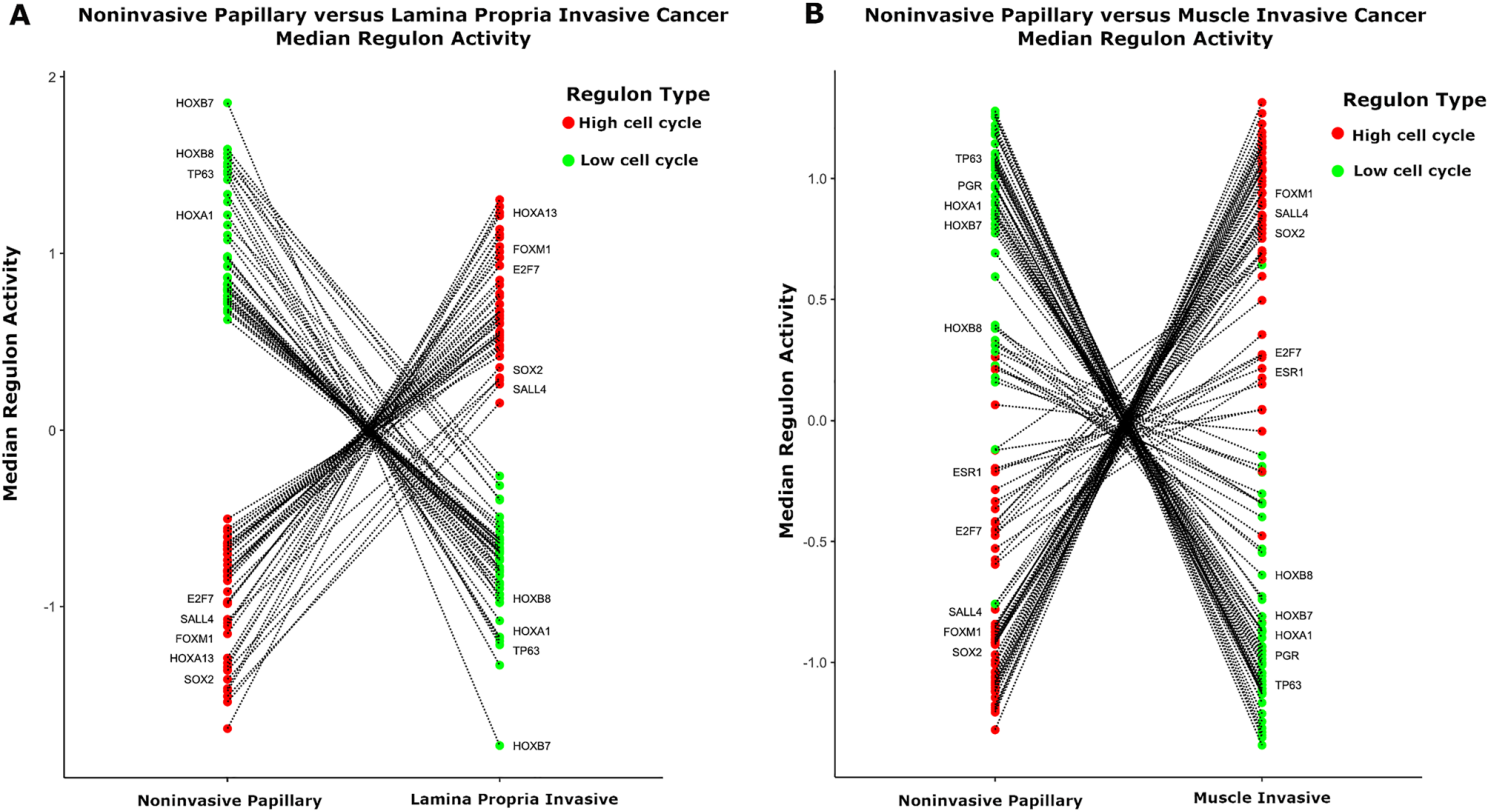
TF regulon activity in noninvasive papillary urothelial carcinoma (NIPUC) versus invasive urothelial carcinoma. (**A**) All 121 TF regulons differed in activity between NIPUC and invasive urothelial carcinoma limited to the lamina propria, all in the expected direction, using the Leeds Cohort (Bonferroni adjusted p < 0.05). (**B**) Similarly, 92% (111 of 121) of TF regulons differed in activity between NIPUC and invasive urothelial carcinoma extending into the muscularis propria, and 93% of these (103 of 111) did so in the expected direction, comparing NIPUC in the UROMOL Cohort to invasive cancers in the TCGA cohort. Both dot plots show regulon activity in NIPUC and invasive cancer, which are in separate columns. Each dot is the median regulon activity for a specific TF. Lines connect the same TF regulon in the NIPUC and invasive groups. Only select TF regulons are labelled, for clarity, and complete results are shown in Table S7.

To test this hypothesis, we first compared activity of the TF regulons between the NIPUC and lamina propria-invasive carcinomas in the Leeds Cohort. This showed all TF regulons differed in activity between NIPUC and lamina propria-invasive cancer, and all did so in the expected directions (Fig. 6A, Bonferroni adjusted p < 0.05, Wilcoxon rank sum test; 112 TF regulons with sufficient data to generate TF regulons). In contrast, no TF regulon differed in activity between NIPUC and lamina propria-invasive cancers in the Uromol Cohort (Bonferroni adjusted > 0.05), a discrepancy without clear cause. We next compared NIPUC in the UROMOL Cohort (i.e. Cohort 2) to MIBC in the TCGA cohort, after normalizing expression levels between the cohorts using EDASeq package^29,30^. This showed 92% (111 of 121) of TF regulons differed in activity between NIPUC and MIBC, and 93% of these (103 of 111) did so in the expected direction (Fig. 6B, Bonferroni adjusted p < 0.05, Wilcoxon rank sum test; full results in Table S7). The findings support that TF regulons in the high cell cycle group are involved in progression of NIPUC to both lamina propria-invasive and muscle-invasive bladder cancer.

## Discussion

In the present study, we identified a transcriptional network active in cell cycle dysregulation of NIPUC. TFs in this network were diverse, and included the pluripotency factors SALL4 and SOX2, classic cell cycle factors such as the E2F family transcription factors, sex steroid binding receptors, and multiple homeobox factors, including HOX factors, POU factors, and SHOX2. Many TFs appeared to act on similar sets of targets, some upregulating and others downregulating them, in keeping with their function in an integrated network. Activity of these TFs largely divided NIPUC into two groups, which differed in activity of the cell cycle. Tumors in the High Cell Cycle group demonstrated greater activity of an established pluripotency signature and higher expression of EZH2 and other members of the PRC2 repressor complex, as well as activity of the pluripotency factors SALL4 and SOX2. The findings suggest these transcription factors induce features of pluripotency in tumors with high cell cycle activity.

There is a link between stem-like properties and EMT in cancer, with the bulk of data indicating tumor cells undergoing EMT become more stem-like^31^. Our data supported this in NIPUC, as our High Cell Cycle tumors demonstrated greater activity of an established EMT signature and greater expression of multiple core EMT transcription factors. However, NIPUC cannot be considered to have undergone EMT by current definitions, which require tumor cells to transition their observable properties to a mesenchymal state, most notably by lost or reduced cell–cell adhesion^20^.

NIPUC are cohesive tumors. They grow from the urothelial surface into the lumen of the bladder as a non-invasive papillary mass, in which the structural integrity of the tumor depends on the tumor cells adhering to one another. These features are the opposite of what is seen in EMT. However, the higher EMT activity seen in High Cell Cycle NIPUC suggests EMT factors may prime NIPUC to convert to a full EMT phenotype and invade. High Cell Cycle NIPUC also demonstrated greater activity of several Rho signatures, which have a well-described role in driving cancer cell motility and invasion^32^, further supporting this idea. Further study will be required to investigate this hypothesis more fully, particularly expression profiling of pared invasive and noninvasive cancer from the same tumor.

We are the first to our knowledge to describe this transcriptional network in NIPUC. However, prior work has been published on salient factors in our analysis, which suggests a relationship among the attributes identified in this study, namely pluripotency, EMT, and HOX factors.

The transcription factors SOX2 and SALL4 were more active in the High Cell Cycle tumors in the present study. These factors are expressed in adult ovary and testis, but are minimally expressed in other normal adult tissues^32–35^. They drive differentiated cells to a pluripotent phenotype, in combination with other pluripotency transcription factors^35–38^. They are also expressed in several cancer types, including bladder cancer, and expression of many of these associates with worse clinical outcomes^36,39^. Both SOX2 and SALL4 drive EMT in tumors^36,40^, and both increase cell proliferation and induce invasion in model systems^36,39^. SOX2 also appears to mark stem-like tumors in bladder cancer^41^. These observations are all consistent with our findings, and suggest SOX2 and SALL4 may be play an important role in driving Stem-like properties, cellular proliferation, and stage-progression of NIPUC. SALL4 is also the target of a therapy in development for hepatocellular carcinoma^42^, suggesting a possible role for this drug in bladder cancer.

The HOX genes were salient in our analysis, accounting for a disproportionate number of significant regulons. HOX genes are best known for their role in coordinating the development of the embryonic body plan^43,44^. These factors also guide growth and renewal of normal adult tissues^44–47^. In cancer biology, HOX genes operate as oncogenes and tumors suppressors^48^. For example, in prostate cancer, HOXC8 acts as an oncogene, while HOXB13 acts as a tumor suppressor gene^48–52^. HOX genes have different effects in different cancer types, highlighting the general principal that TFs act in a context-dependent manner. As an example, HOXB13 acts as an oncogene in breast cancer^53^, opposite to its effect in prostate cancer. HOXB13 is part of a biomarker test currently used in breast cancer^54^, highlighting the potential value of HOX factors as tumor biomarkers. HOX genes have been previously described in bladder cancer as well, with findings consistent with those in the current study. For example, it has been shown that HOXB factors and anterior HOXA factors are expressed in both normal urothelium and urothelial carcinomas with less aggressive features, while cancers with more aggressive features lack expression of these HOX factors^55–57^.

The sex steroid binding receptors ESR1 and PGR were also salient in our analysis, the former active in High Cell Cycle NIPUC and invasive cancer, and the latter active in Low Cell Cycle NIPUC and non-invasive cancer. The former findings are consistent with prior studies. For example, it has been shown ESR1 expression is elevated in high grade and high stage urothelial carcinoma^58–60^, and expression of ESR positively correlates with cell cycle activity^60^. The findings suggest ESR1 may also be a therapeutic target of NIPUC.

Our findings also suggest that changes in chromatin state may influence activity of the transcription factors we identified. “Chromatin state” describes the openness or closedness of chromatin, which is controlled by epigenetic modifications, such as histone methylation. An important histone methylase is PRC2, a multiprotein complex that induces a closed chromatin configuration that silences associated genes^61^. This complex plays a vital role in embryogenesis by silencing genes that drive differentiation, maintaining cells in a pluripotent state^61^. Members of the PRC2 complex, including EZH2, are expressed in a subset of cancers, including bladder cancer^23^. Consistent with its role in embryogenesis, the PRC2 complex inhibits cellular differentiation and drives cellular proliferation in cancer, partly through histone methylation^62^. Furthermore, EZH2 induces expression of pluripotency factors, such as SOX2, in a subset of cancers^63^, indicating a possible driver of TF expression. The combined findings suggest the PRC2 complex plays a role in governing the transcriptional network identified in the present study. EZH2 is also the target of tazemetostat, a small molecule inhibitor effective in treating follicular lymphoma and epithelioid sarcoma^64,65^, suggesting a potential role for the drug in bladder cancer.

The present study also showed greater mutational burden and genomic instability in NIPUC with higher cell cycle activity, the latter finding consistent with prior studies^26^. However, a specific mutational cause of cell cycle dysregulation, such as homozygous *CDKN2A* loss, was identified in only half of High Cell Cycle cases. The findings suggest activity of TFs may play a direct role in driving cell cycle activity in these tumors, possibly through epigenetic mechanisms.

In conclusion, we have identified a transcriptional regulatory network operative in cell cycle dysregulation both in invasive and non-invasive bladder cancer. HOX and pluripotency factors appear to play a prominent role. The findings offer insights into the biological processes that drive clinical behavior in the disease, and may help guide development of targeted therapy and diagnostic biomarkers.

## Materials and methods

### Methods details

Our study was retrospective and utilized three cohorts of NIPUC. These were used to develop the transcriptional network and test its relationship with biological features, such as pathway activity. Cohort 1 consisted of NIPUC on which whole transcriptome RNA sequencing, whole exome DNA sequencing, and immunohistochemistry were newly performed, specifically for this study (n = 81, RNA sequencing; DNA sequencing and immunohistochemistry for EZH2 on 36 and 21 of these, respectively). This cohort was studied with approval from the Penn State College of Medicine Human Subjects Protection Office (Institutional Review Board). Cohort 2 consisted of cases of NIPUC from the UROMOL study, for which RNA sequencing-based expression data are publicly available (n = 397)^12^. Cohort 3 consisted of cases of NIPUC from Leeds University, for which expression data by Affymetrix microarray are publicly available (n = 107).^13^

We also compared activity of the transcriptional network between NIPUC and invasive urothelial carcinoma. This included both invasive cancers limited to the lamina propria and those invading the muscularis propria. This was performed using lamina propria-invasive cancers from the Uromol (n = 135)^12^ and Leeds (n = 102)^13^ Cohorts, and MIBC from The Cancer Genome Atlas (TCGA) cohort^14^.

Our approach utilized Cohorts 1 and 2 to robustly develop the TF regulons, and Cohort 3 to validate them. We used Cohort 2 for dual regulon analysis and pathway analysis, given the greater statistical power conferred by the large cohort size. Comparison between invasive and noninvasive cancers was performed on single cohorts, specifically the broader (i.e. NIPUC plus lamina propria invasive cancers) UROMOL Cohort and the Leeds Cohort individually, and the cohorts in which the same material and methodology were used, specifically the TCGA cohort and UROMOL cohort, both of which were performed from fresh frozen tissue using RNA sequencing.

### Approach to multiple testing adjustments

Our analysis required considered use of multiple testing adjustments. We chose FDR in situations in which sensitivity was paramount, such as establishing regulons and performing pathway analysis. We chose Bonferroni adjustment where a targeted questions was asked, specifically which regulons differ in activity between invasive and noninvasive cancers. We did not adjust for multiple testing if 5 or fewer selected variables were assessed, such as the five canonical EMT TFs. We included the 12 most commonly mutated genes in multiple testing correcting for comparison of individual gene mutations in High Cell Cycle and Low Cell Cycle tumors.

### RNA sequencing

For Cohort 1, total (rRNA depleted) RNA sequencing was performed from formalin fixed paraffin embedded (FFPE) tissue. Tumor tissue was macrodissected from 10 micron unstained slides, then extracted using Qiagen FFPE kits (Venlo, Netherlands). RNA-sequencing libraries were generated using KAPA RNA HyperPrep Kits with RiboErase (HMR) (Roche). The unique dual index sequences (NEXTFLEX® Unique Dual Index Barcodes, BioO Scientific) were incorporated in the adaptors for multiplexed high-throughput sequencing. The final product was assessed for its size distribution and concentration using BioAnalyzer High Sensitivity DNA Kit (Agilent Technologies). The libraries were pooled and diluted to 3 nM using 10 mM Tris–HCl, pH 8.5 and then denatured using the Illumina protocol. The denatured libraries were loaded onto an S1 flow cell on an Illumina NovaSeq 6000 (Illumina) and run for 2X50 cycles according to the manufacturer's instructions. De-multiplexed and adapter-trimmed sequencing reads were generated using Illumina bcl2fastq (released version 2.18.0.12) allowing no mismatches in the index read. BBDuk (https://jgi.doe.gov) was used to trim/filter low quality sequences using “qtrim = lr trimq = 10 maq = 10” option. Next, alignment of the filtered reads to the human reference genome (GRCh38) was done using HISAT2 (version 2.1.0) (https://ccb.jhu.edu/software/hisat2/manual.shtml) applying –no-mixed and –no-discordant options. Read counts were calculated using HTSeq^66^ by supplementing Ensembl gene annotation (GRCh38.78). Only cases with alignment rate > 85% were included in downstream analysis. Expression was estimated from read counts using FPKM, via the edgeR package^67^, then log2 transformed. RNA sequencing on Cohort 2 is previously described^11^. The expression matrices for Cohort 1 and Cohort 2 were restricted to genes annotated in both data sets.

### Expression analysis of the Leeds cohort

The Leeds Cohort comprised 102 stage lamina propria-invasive (stage T1) and 107 NIPUC (stage Ta) tumors from the Leeds Multidisciplinary Research Tissue Bank (REC reference: 10/H1306/7). Cohort 3 comprised the NIPUC from this cohort. Total RNA was isolated from frozen tissue sections containing ≥ 70% tumor cells using a RNeasy Plus Micro Kit and amplified using the Affymetrix GeneChip WT PLUS Reagent Kit. The resulting cDNA was quantified using OD, normalized and hybridized onto Affymetirx Human Transcriptome 2.0 microarrays. Microarrays were washed and stained using Affymetrix GeneChip Hyridisation, Wash and Stain Kit. Quality control checks, gene level normalization (using SST-RMA) and signal summarization was conducted using Affymetrix Expression console software.

### Clustering into early vs late cell cycle groups

To assign tumors into early versus late cell cycle groups, tumors were clustered using an established list of cell cycle genes^11^ and 2-means (k clustering with a 2-cluster solution) consensus clustering, applied to RNA sequencing data from Cohort 1, Cohort 2, and Cohort 3, separately. ConsensusClusterPlus^68^ was utilized, with 90% item resampling and 100% feature resampling, Euclidean distance, and 1000 repetitions.

### Generating TF regulons and a transcriptional network

We first clustered NIPUC from Cohort 2 into early versus late cell cycle groups, the latter considered to have greater cell cycle activity, via consensus clustering using an established list of cell cycle genes^11^. Clustering placed 192 cases into the early cell group and 205 into the late cell cycle group. We then generated a transcriptional regulatory network from expression data using the ARACNe algorithm, a method that takes a list of curated TFs and identifies target genes likely regulated by each of them, using mutual information between expression of a given TF and potential target genes^14–17^, and the RTN package^15^. The targets of each TF are termed its “TF regulon.” We used a list of 1639 curated TFs^18^. We then used a step-wise approach with two layers of statistical tests, and found 413 of these TF regulons differed in activity between early and late cell cycle groups (first layer = hypergeometric test, second layer = Wilcoxon sum test applied; FDR adjusted p < 0.05 considered significant; only TF regulons significant on both layers of analysis were included; details of approach shown Fig. S1). To winnow this list to the TF regulons most strongly associated with cell cycle dysregulation, we utilized Cohort 1 to investigate the association between activity of these 413 TF regulons and two measures of cell cycle activity: cell cycle group and mitotic index (the number of mitotic figures per 10 high-powered microscopic fields, which served as an orthogonal measure of cell cycle dysregulation)^4^. Cell cycle group was assigned by consensus clustering, using the approach described above. Analysis of the Cohort 1 was based on single sample GSEA enrichment scores, which were determined for each TF regulon in each sample using GSEA2 function in RTN^15^. Activity of each TF regulon was quantified in each tumor as “enrichment score,” a continuous variable reflecting expression of targets within each TF regulon, determined using single sample GSEA (highly positive scores indicate high enrichment, highly negative score indicate low enrichment; enrichment scores ranged from − 2 to 2)^15^. Relative enrichment of regulons in the late versus early cell cycle groups was quantified as “enrichment score difference,” the median enrichment score in the late cell group minus the median enrichment score in the early cell cycle group, determined separately for each TF regulon.

### TF regulon activity and signatures of cellular signaling, pluripotent stem cells, and epithelial-mesenchymal transition

This portion of the analysis used NIPUC tumors from Cohort 1 and Cohort 2, separately. Samples were clustered based on activity of the 121 significant TF regulons, using ConsensusClusterPlus, with k-means clustering, 90% item resampling, 100% feature resampling, Euclidean distance, and 1000 repetitions. A two-cluster solution was chosen based on principal component analysis (PCA) and consensus cumulative distribution function (CDF) analysis^68,69^. In PCA, TF regulon activity was reduced to two principal components, and visually examined to identify best grouping of samples. In consensus CDF curve analysis, the curve with the smallest number of clusters and greatest area under the curve was chosen. Both led to a two-cluster solution as most reasonable. Clusters were named “High Cell Cycle” and “Low Cell Cycle.” Pathway analysis was performed using the GSEA graphical user interface, on samples from Cohort 2^68–72^. The Reactome gene set database was utilized (807 total gene lists evaluated). Reactome gene lists were considered significant if they demonstrated significant enrichment with q < 0.05, False Discovery Rate.

To compare TF regulon activity to signatures of EMT and pluripotency, we quantified activity of EMT and pluripotency in each sample using RNA sequencing expression data, single sample GSEA via the GSVA package in Bioconductor^73^, and gene lists of EMT^21^ and cancer cell pluripotency (“ES Exp2” in^20^). Activity of these EMT and pluripotency signatures was then compared among the High Cell Cycle and Low Cell Cycle groups described above using the Wilcoxon rank sum test. We also evaluated differences between High and Low Cell Cycle tumors in expression of individual core EMT transcriptions factors (*SNAI1*, *SNAI2*, *ZEB1*, *ZEB2*, *TWIST*) and members of the PRC2 complex (*SUZ12*, *EZH1*, *EZH2*, *EED*), using RNA sequencing data. We also evaluated expression of EZH2 at the protein level by immunohistochemistry in 21 NIPUC from Cohort 1, wherein differences between High and Low Cell Cycle tumors were evaluated with the Wilcoxon rank sum test.

### Immunohistochemistry

EZH2 immunohistochemistry was performed using a Ventana autostainer and antibody from Leica Microsystems, #NCL-L-EZH2. Retrieval: 48 min. Antibody 1:100 (made in ADB250) incubated for 1 h @36C. anti-mouse HRP multimer, Roche, #760-4310 for 20 min.

### Dual regulons

We used the RTNduals package^74^ to test the association between pairs of TF regulons, using Fisher’s exact test to assess the statistical significance of the overlap between regulons, and Pearson’s correlation to assign the direction of association (i.e. positive or negative).

### Comparison between NIPUC, lamina propria-invasive cancer, and MIBC

We compared TF regulon activity in NIPUC to both lamina propria-invasive bladder cancer and MIBC. This was done with three separate groups of tumors: (1) NIPUC and lamina propria-invasive cancers from the Leeds Cohort, which required no normalization; (2) NIPUC and lamina propria-invasive cancers from the UROMOL Cohort, which required no normalization; and (3) NIPUC from the UROMOLL Cohort combined with MIBC from the TCGA Cohort, which did required normalization. NIPUC where compared to lamina propria-invasive cancers in the Leeds Cohort using the Wilcoxon rank sum test (Bonferroni adjusted p < 0.05 considered significant). NIPUC where likewise compared to lamina propria-invasive cancers in the UROMOL Cohort using the Wilcoxon rank sum test (Bonferroni adjusted p < 0.05 considered significant). Gene expression data from NIPUC from the UROMOL Cohort were normalized to expression data from the TCGA cohort, as detailed below, using an established approach. The validity of this normalization approach was considered affirmed by consistency with findings in the cohorts that did not require normalization.

To compare gene expression data between NIPUC in the UROMOL Cohort to MIBC in the TCGA Cohort, the TCGAbiolinks R package^73–77^ was used to download HT-Seq gene-level read counts for the TCGA BLCA cohort. The resulting data set was then restricted to protein coding genes and primary tumor samples. Digitally scanned slides from the TCGA bladder cancer cases were reviewed by a urologic pathologist (JIW) using the digital archive, and cases were identified that demonstrate only NIPUC (Table S9). These cases were excluded from this portion of the analysis (and were used for copy number analysis, below). After restricting to a common set of 19,012 genes, the HT-Seq read counts from the TCGA cohort were merged with the Salmon read counts from NIPUC from the UROMOL Cohort. The EDASeq R package^29^ was used to create a SeqExpressionSet based on the combined read counts, then upper quantile normalization was performed. The RUVSeq R package^30^ was then applied using k = 1 and a set of eleven housekeep genes described by Eisenberg and Levanon^78^ to identify a covariate corresponding to factors of unwanted variation. This covariate was then used to produce normalized counts that were corrected for the factors of unwanted variation. Exploratory analyses (PCA plots, unsupervised clustering, etc.) performed on the normalized counts strongly suggested that there was no underlying batch effect in the merged data set. The normalized counts were then converted to transcript per million (TPM) values. Activity of each of the 121 TF regulons was quantified in each tumor using single sample GSEA, via the tni.replace.samples function in RTN. Activity of NIPUC from the UROMOL Cohort were then compared with MIBC from the TCGA cohort, using the Wilcoxon rank sum test (Bonferroni adjusted p < 0.05 considered significant).

### DNA Sequencing and copy number analysis

DNA sequencing was performed from formalin fixed paraffin embedded (FFPE) tissue on 36 patients from Cohort 1. Tumor tissue was macrodissected from 10 micron unstained slides, then extracted using Qiagen FFPE kits (Venlo, Netherlands). The samples were sequenced using a 2 × 150 bp Paired End (PE) configuration. Image analysis and base calling were conducted by the HiSeq Control Software (HCS). Raw sequence data (.bcl files) generated from Illumina HiSeq was converted into fastq files and de-multiplexed using Illumina's bcl2fastq 2.17 software. One mismatch was allowed for index sequence identification. GRCH37/HG19 was used as reference. To filter spurious calls from FFPE artifact, we included only mutational base calls with depth greater than 50, allelic fraction greater than 5%, and number of mutated reads greater than 10, similar to recommendations by the Association for Molecular Pathology and College of American Pathologists^64^. Tumor mutational burden was defined as total number of mutations after filtering, and all tumors were sequenced with the same protocol and identical target size.

Copy number analysis was performed using segmentation files produced by the CopywriteR package^79^, which generates copy number levels on a log ratio scale, giving values that are zero for copy number neutral, positive for copy number gains, and negative for copy number losses. Thresholds for copy number alterations were based on segmentation mean quantiles from RNA sequencing-based expression data from TCGA cases in which only NIPUC was sampled (see above and Table S9). Specifically, we assembled thresholded and continuous segmentation value copy number data on these cases using TCGAbiolinks. We then divided the segmentation loci into quantiles based on the continuous segmentation copy number value. We then identified the quantiles corresponding to threshold values, specifically high and low gains (i.e. 2 and 1) and heterozygous and homozygous losses (i.e. − 1 and − 2). We then turned to our cases, and divided segmentation loci into quantiles based on continuous copy number values, and assigned threshold values based on the quantiles identified in using the TCGA data. Using this approach, copy number gains were set at segmentation mean > 0.1, with high copy number gain set at > 1.05. Copy number losses were set at segmentation mean < − 0.1, with homozygous loss set at < − 0.75. Genomic instability was defined as the mean of chromosome-level segmentation means for each sample, similar to prior studies^28^. Specifically, for each tumor we took the mean of the absolute value of the segmentation means for each chromosome. We then took the mean of these chromosome-level values, and defined Genomic Instability as that number.

## Supplementary Information

Supplementary Legends.

Supplementary Legends.

Supplementary Figure S1.

Supplementary Figure S2.

Supplementary Figure S3.

Supplementary Figure S4.

Supplementary Figure S5.

Supplementary Figure S6.

Supplementary Table S1.

Supplementary Table S2.

Supplementary Table S3.

Supplementary Table S4.

Supplementary Table S5.

Supplementary Table S6.

Supplementary Table S7.

Supplementary Table S8.

Supplementary Table S9.

## Data Availability

Data are available for all datasets in this paper: Cohort 1 (log2 (x + 1) normalized expression data in Table S8), Cohort 2 (EGAS00001004693), and Cohort 3 (GSE163209). The TCGA cohort is available through the GDC data portal (https://portal.gdc.cancer.gov) and TCGAbiolinks^75^.
