# Supplementary material for: A transcriptional network of cell cycle dysregulation in noninvasive papillary urothelial carcinoma
Source: Sci Rep. 2022 Oct 3;12:16538. doi: 10.1038/s41598-022-20927-9 (PMC9529892; doi:10.1038/s41598-022-20927-9)
Supplement: Supplementary file 1 — Supplementary Legends. [file 41598_2022_20927_MOESM1_ESM.docx]

**Supplemental figure captions**

**Figure S1:** A schematic describing the method for selecting significant TF regulons in our analysis.

**Figure S2:** Differences between the High Cell Cycle and Low Cell Cycle groups in expression of core TFs of EMT. Red indicates p<0.05 (Wilcoxon rank sum test).

**Figure S3:** EZH2 expression in NIPUC by immunohistochemistry (200X magnification)

**Figure S4**: Differences between the High Cell Cycle and Low Cell Cycle groups in expression of members of the PRC2 complex. All were statistically significant, p<0.05 (Wilcoxon rank sum test).

**Figure S5**: High Cell Cycle tumors, as determine by clustering with TF regulon activity, were enriched in Class 2a or 2b tumors, while Low Cell Cycle tumors tended to be either Class 1 or 3, as defined by Lindskrog et al (12) (p<0.001, Fisher’s exact test).

**Figure S6**: *CDKN2A* copy number losses and gene expression at the RNA level. Cases with homozygous loss demonstrated significantly lower gene expression compared with those showing no loss or heterozygous loss. Numbers over brackets between boxes indicate p-values, calculated using the Wilcoxon rank sum test.
