# Supplementary material for: A transcriptional network of cell cycle dysregulation in noninvasive papillary urothelial carcinoma
Source: Sci Rep. 2022 Oct 3;12:16538. doi: 10.1038/s41598-022-20927-9 (PMC9529892; doi:10.1038/s41598-022-20927-9)
Supplement: Supplementary file 2 — Supplementary Legends. [file 41598_2022_20927_MOESM2_ESM.docx]

**Supplemental table captions**

**Table S1:** Clinical details of patients in Cohorts 1, 2, and 3.

**Table S2.** Transcription factor regulons. Shown is each of the 121 transcription factor regulons significant on all layers of analysis detailed in Figure S1. Each column is a regulon; the column name is the transcription factor and all rows beneath are its targets.

**Table S3**. Transcription factor regulon associations with cell cycle group in Cohort 3. Direction in late cell cycle refers to the median enrichment score (per ssGSEA) in the late cell cycle group minus the median enrichment score in the early cell cycle group. P-values were calculated with the Wilcoxon rank sum test.

**Table S4**. Complete results of unsupervised GSEA analysis using Cohort 2 and Reactome gene lists. The two groups compared are High Cell Cycle and Low Cell Cycle, assigned by clustering tumors by regulon activity.

**Table S5.** Dual regulon analysis performed on Cohort 2. R.regulons indicates association between regulon activity. P-values were calculated by Spearman correlation.

**Table S6**. Complete list of somatic mutations identified in 36 sequenced tumors from Cohort 1.

**Table S7.** Enrichment score differences between invasive urothelial carcinoma and noninvasive papillary urothelial carcinoma, using the UROMOL/TCGA Cohorts (A) and the Leeds Cohort (B). Direction in invasive (either MIBC or lamina propria invasive (T1)) refers to the median enrichment score (per ssGSEA) in the invasive group minus the median enrichment score in the noninvasive papillary urothelial carcinoma group. P-values were calculated with the Wilcoxon rank sum test. MIBC=muscle invasive bladder cancer, T1=lamina propria-invasive cancer; Ta=noninvasive papillary urothelial carcinoma. Agree=enrichment score difference was in the expected direction. Adj.p=Bonferroni adjusted p-value.

**Table S8**. Expression matrix of tumors from Cohort 1. All values are log2(x+1) transformed.

**Table S9**. Tumors in the TCGA data set in which only noninvasive papillary carcinoma was sampled, with no clear invasive carcinoma. Digital slides reviewed by the study pathologist (JIW).
