# Supplementary figures and images for: A transcriptional network of cell cycle dysregulation in noninvasive papillary urothelial carcinoma
Source: Sci Rep. 2022 Oct 3;12:16538. doi: 10.1038/s41598-022-20927-9 (PMC9529892; doi:10.1038/s41598-022-20927-9)

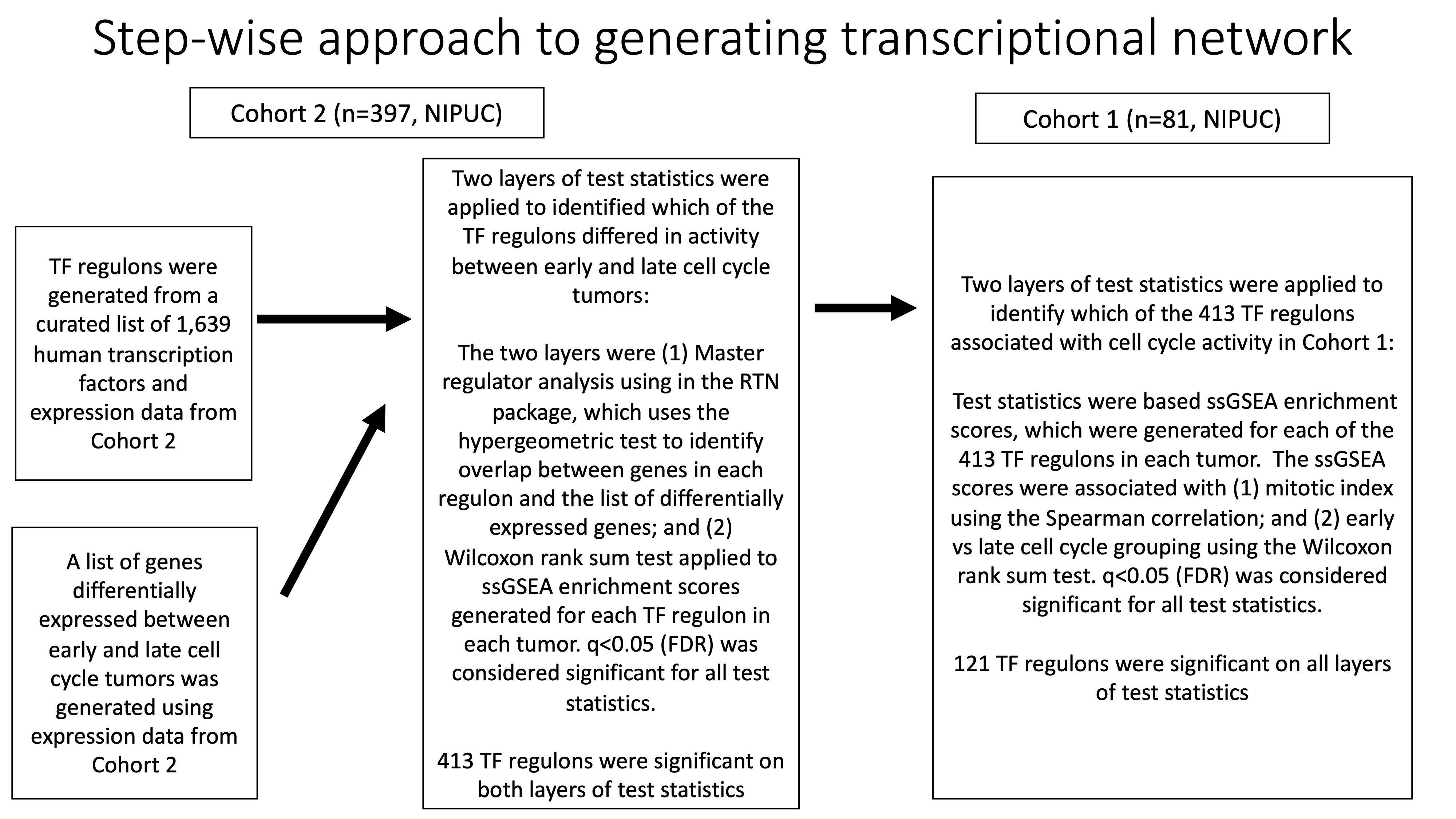

Supplement: Supplementary file 3 — Supplementary Figure S1. [file 41598_2022_20927_MOESM3_ESM.tiff]

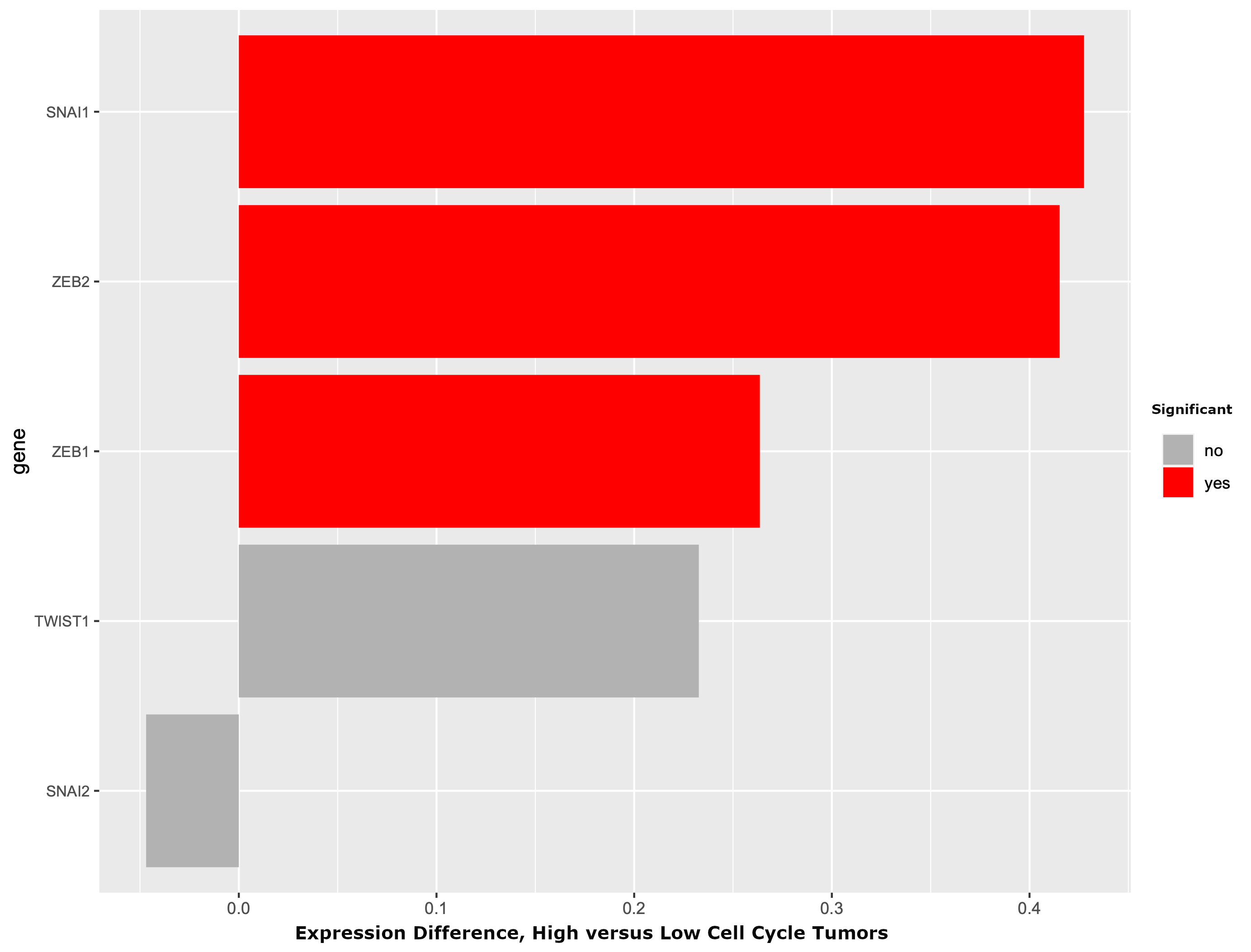

Supplement: Supplementary file 4 — Supplementary Figure S2. [file 41598_2022_20927_MOESM4_ESM.tiff]

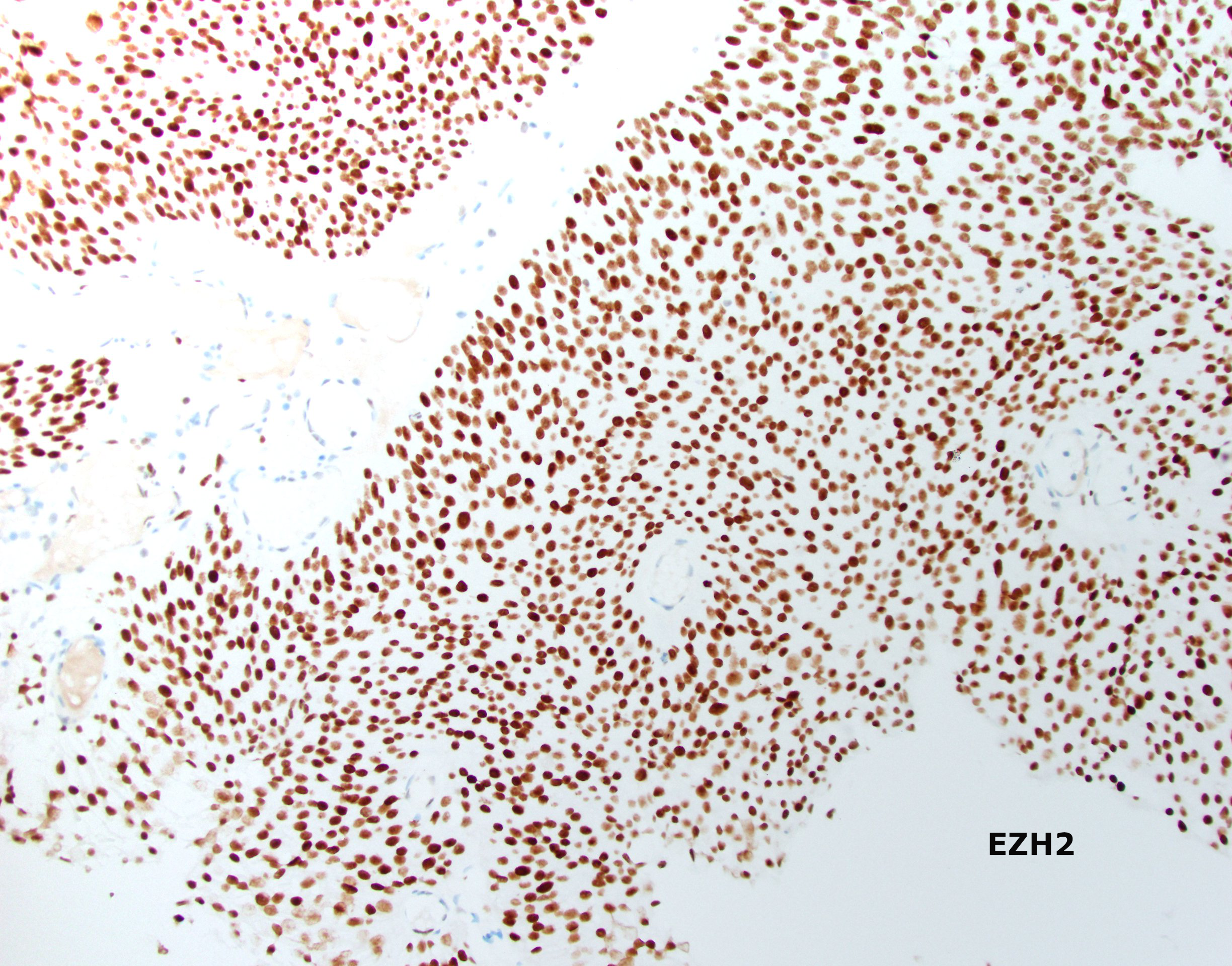

Supplement: Supplementary file 5 — Supplementary Figure S3. [file 41598_2022_20927_MOESM5_ESM.tiff]

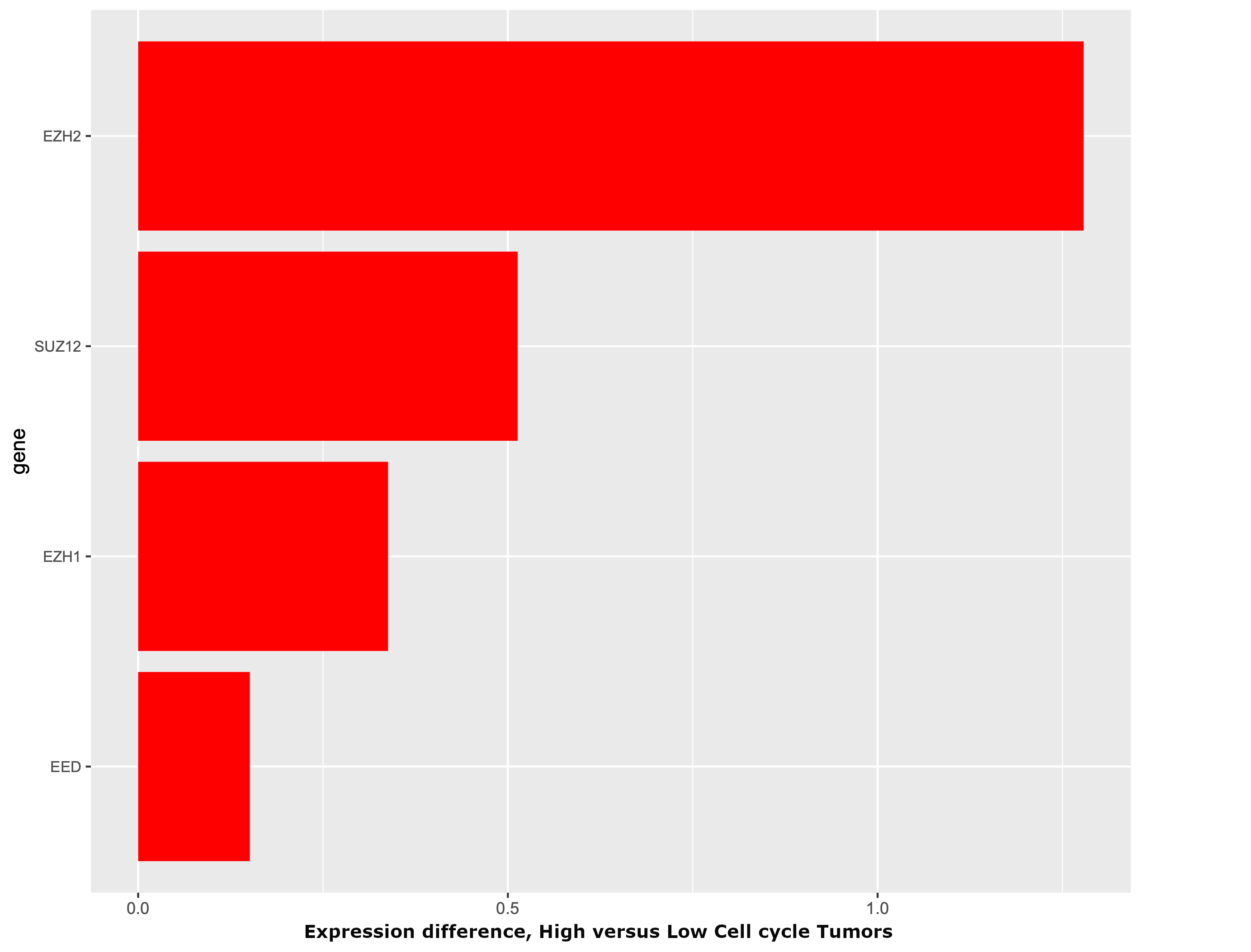

Supplement: Supplementary file 6 — Supplementary Figure S4. [file 41598_2022_20927_MOESM6_ESM.tiff]

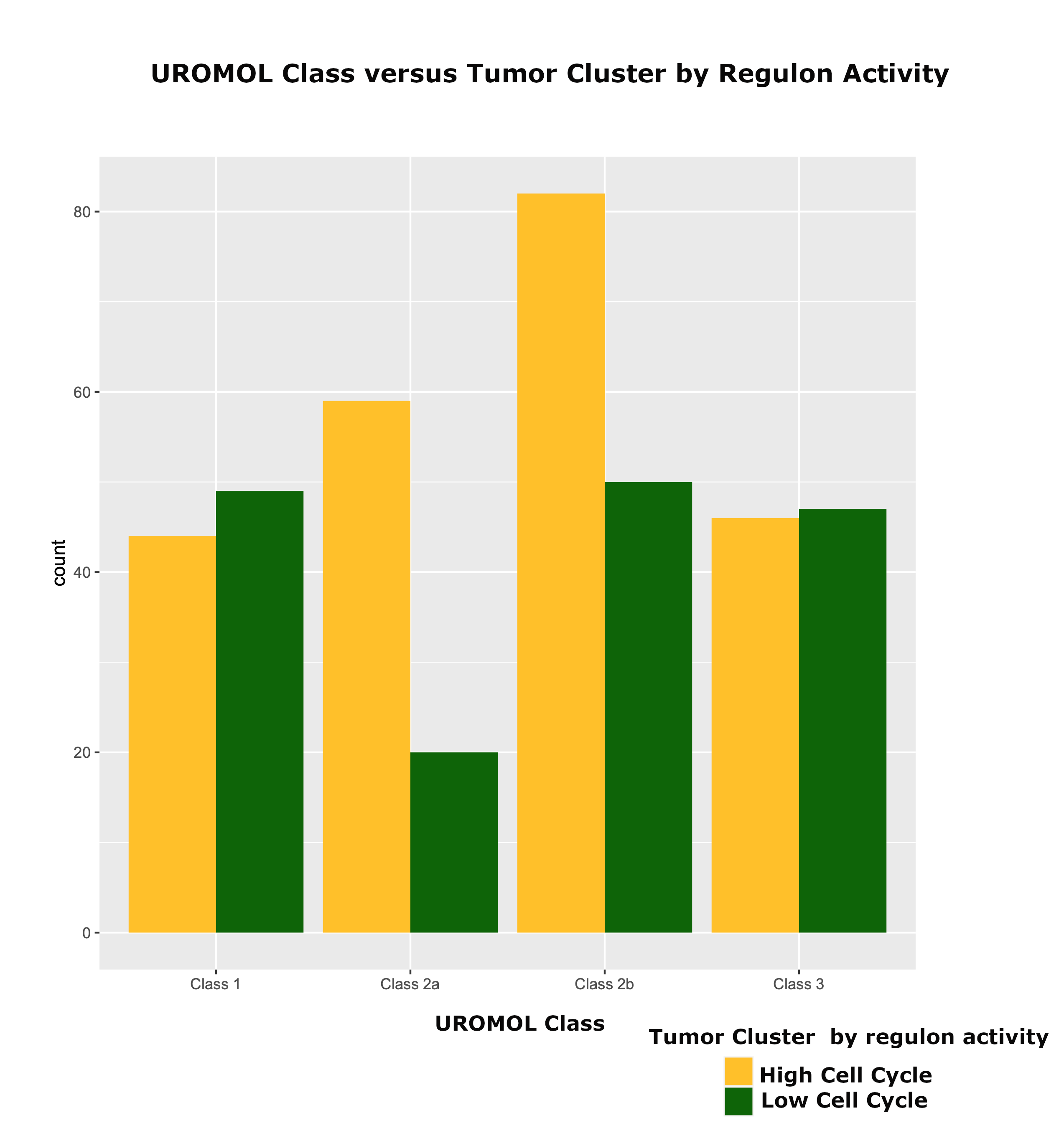

Supplement: Supplementary file 7 — Supplementary Figure S5. [file 41598_2022_20927_MOESM7_ESM.tiff]

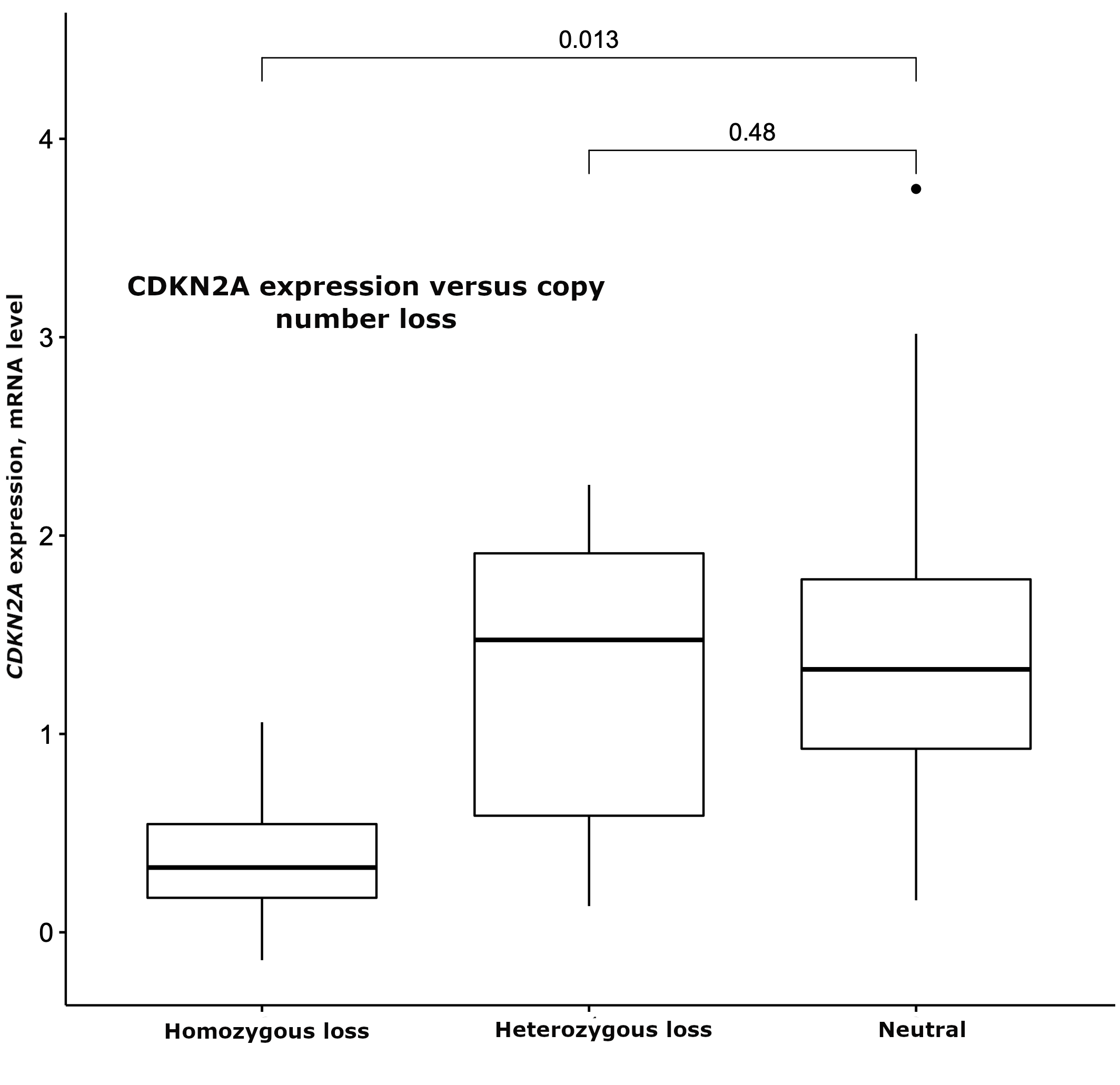

Supplement: Supplementary file 8 — Supplementary Figure S6. [file 41598_2022_20927_MOESM8_ESM.tiff]
