# Supplementary material for: A transcriptional network of cell cycle dysregulation in noninvasive papillary urothelial carcinoma
Source: Sci Rep. 2022 Oct 3;12:16538. doi: 10.1038/s41598-022-20927-9 (PMC9529892; doi:10.1038/s41598-022-20927-9)
Supplement: Supplementary file 9 — Supplementary Table S1. [file 41598_2022_20927_MOESM9_ESM.docx]

| **Table S1:** Cohort details, including number of subject (n) by stage, and percent of patients experiencing recurrence and progression | | | | | | | | | | | |
| --- | --- | --- | --- | --- | --- | --- | --- | --- | --- | --- | --- |
| Cohort 1 | | | | Cohort 2 | | | | Cohort 3 | | | |
| n | Recurrence | Progression | High-grade | n | Recurrence | Progression | High-grade | n | Recurrence | Progression | High-grade |
| 81 | 48% | 5% | 28% | 345 | 64% | 7% | 33% | 107 | 45% | 1% | NA |
